# Function of Macrophage and Parasite Phosphatases in Leishmaniasis

**DOI:** 10.3389/fimmu.2017.01838

**Published:** 2017-12-22

**Authors:** Didier Soulat, Christian Bogdan

**Affiliations:** ^1^Mikrobiologisches Institut – Klinische Mikrobiologie, Immunologie und Hygiene, Universitätsklinikum Erlangen, Friedrich-Alexander-Universität (FAU) Erlangen-Nürnberg, Erlangen, Germany; ^2^Medical Immunology Campus Erlangen, Interdisciplinary Center of the FAU, Erlangen, Germany

**Keywords:** *Leishmania*, macrophages, protein tyrosine phosphatase, signaling, exosome

## Abstract

The kinetoplastid protozoan parasites belonging to the genus *Leishmania* are the causative agents of different clinical forms of leishmaniasis, a vector-borne infectious disease with worldwide prevalence. The protective host immune response against *Leishmania* parasites relies on myeloid cells such as dendritic cells and macrophages in which upon stimulation by cytokines (e.g., interferon-γ) a complex network of signaling pathways is switched on leading to strong antimicrobial activities directed against the intracellular parasite stage. The regulation of these pathways classically depends on post-translational modifications of proteins, with phosphorylation events playing a cardinal role. *Leishmania* parasites deactivate their phagocytic host cells by inducing specific mammalian phosphatases that are capable to impede signaling. On the other hand, there is now also evidence that *Leishmania* spp. themselves express phosphatases that might target host cell molecules and thereby facilitate the intracellular survival of the parasite. This review will present an overview on the modulation of host phosphatases by *Leishmania* parasites as well as on the known families of *Leishmania* phosphatases and their possible function as virulence factors. A more detailed understanding of the role of phosphatases in *Leishmania*–host cell interactions might open new avenues for the treatment of non-healing, progressive forms of leishmaniasis.

## Introduction

### Epidemiology and Disease Development

Leishmaniasis is a worldwide prevalent infectious disease. Among all parasitic infections, leishmaniasis ranks second in mortality after malaria (1). Leishmaniasis is primarily encountered in tropical and subtropical countries, with an estimated number of 0.7–1 million new cases of cutaneous leishmaniasis (CL) and 50,000 to 90,000 new cases of visceral leishmaniasis accompanied by 20,000–30,000 deaths per year (World Health Organization^1^ [WHO]). Due to the absence of an effective and well-tolerated vaccine, the lack of simple and efficient treatments and the strong correlation between disease and poor socioeconomic conditions, WHO has classified leishmaniasis as one of the 20 neglected tropical diseases worldwide.^2^

Leishmaniasis is caused by kinetoplastid parasites of the Trypanosomatidae family that comprises monogenetic genera often found in insects or plants as well as several human pathogens of the heteroxenous genera *Trypanosoma* and *Leishmania* (*L*.). In nature, *Leishmania* parasites are transmitted by the bites of sand flies, which inject the extracellular, flagellated parasite stage (so-called infective or metacyclic promastigote) into the dermis of the skin. Once promastigotes are released into the blood pool of the skin wound, they are endocytosed by phagocytic cells (initially polymorphonuclear, neutrophilic granulocytes [PMNs], then blood monocytes, dendritic cells, and macrophages) in which they differentiate into intracellular, so-called amastigote *Leishmania* (2–4). Amastigotes contain only a stumpy rest of the flagellum within the flagellar pocket of the parasite. *Leishmania* amastigotes will multiply in non-activated phagocytic cells and are transported *via* the lymph and blood stream to various tissues and organs (e.g., draining lymph nodes, spleen, liver, bone marrow, healthy skin), depending on the host immune response and the *Leishmania* species involved. The life cycle of the parasite will be completed once a sand fly ingests amastigote-infected tissue phagocytes during its blood meal. Within the digestive tract of the vector, the parasites retransform into promastigotes, which develop from a non-infective procyclic into the infective metacyclic stage (5).

Depending on the affected tissues, leishmaniasis can be divided into three major forms: CL, mucocutaneous leishmaniasis (MCL), and visceral leishmaniasis (VL, also known as Kala-Azar) (6). Members of the subgenus *Leishmania* (e.g., *L. major, L. tropica, L. infantum, L. mexicana, L. amazonensis*) and the subgenus Viannia (*L. braziliensis, L. guyanensis, L. panamensis)* can cause cutaneous disease. CL is characterized by chronic papular, erythematous, plaque-like, or ulcerative skin lesions. These lesions ultimately heal in immunocompetent patients after months to years of infection (7). MCL symptoms develop especially after an infection with the South American species *L. braziliensis, L. panamensis* or *L. guyanensis*. This form of the disease is usually associated with a higher morbidity because of the destruction of mucocutaneous tissue sites such as the nose and nasal septum, the oropharynx or the palate (7). VL is the most severe form of the disease due to the systemic spread of the parasite and usually results from an infection with viscerotropic *L. infantum* strains (identical with *Leishmania chagasi*) or *Leishmania donovani*. VL, which is characterized by persistent fever, enlargement of liver and spleen, and pancytopenia resulting from secondary hemophagocytosis in the bone marrow, is lethal if untreated (8, 9). All these various disease developments are the result of a complex equation influenced by the arsenal of virulence factors of the parasite and the immune status of the host (10).

### Innate Immune Response against *Leishmania* and Importance of Phosphorylation Events: A Brief Overview

To survive during the infection of mammalian hosts, *Leishmania* parasites must quickly adapt to their new and hostile environment. Expression of the stress response protein A2 specifically by *Leishmania* species causing VL is a good example of such adaptation. Indeed, A2 promotes heat shock resistance and thereby supports parasite survival and visceralization in inner and warmer organs (11). However, *Leishmania* parasites could not survive in their new host organisms without the development of additional strategies to escape the host defense mechanisms.

Phagocytic cells, such as neutrophils, dermal dendritic cells, and dermal macrophages, are the first immune cells encountered by the promastigotes injected into the skin by sand flies. In addition to being a primary source of antileishmanial effector molecules, they also represent the cellular niche chosen by *Leishmania* promastigotes to evade the cytotoxic humoral components (such as complement) and/or to differentiate into replicating amastigotes (12–14). Therefore, it is crucial to understand how *Leishmania* convert these microbicidal phagocytes into safe target cells.

Uptake of pathogens by phagocytes usually results in a proinflammatory innate immune response, which fosters their microbicidal activity and promotes the recruitment and activation of other immune cells. In the case of *Leishmania* parasites, PMNs are the first line of defense and exert protective effects *via* release of reactive oxygen species (ROS) and the formation of neutrophil extracellular traps. At the same time *Leishmania*-infected apoptotic PMNs also function as deactivating vehicles for the transfer of the parasites into macrophages and dendritic cells [reviewed in Refs. (2, 15, 16)]. Subsequently, macrophages and dendritic cells become activated by interferon (IFN)-γ and tumor necrosis factor (TNF) generated by natural killer cells and CD4^+^ T lymphocytes, which leads to the expression of inducible or type 2 nitric oxide (NO) synthase (iNOS, NOS2) [reviewed in Ref. (17)]. iNOS-derived NO is not only a central antileishmanial effector molecule (18–21) but also paves the ensuing type 1 T helper (Th1) cell response [reviewed in Ref. (22)]. All cellular signaling pathways engaged in this response are tightly controlled and depend on numerous posttranslational protein modifications. Among them, versatile phosphorylation of molecules plays major and ubiquitous functions. It is therefore not a surprise that *Leishmania* parasites have been described to manipulate such a cardinal modification to attenuate the host immune response.

Phagocytosis is the first action undertaken by macrophages against transmitted *Leishmania*. However, maturation of the phagosome toward a lytic phagolysosome can be altered by one of the most abundant virulence factors produced by *Leishmania* promastigotes, the lipophosphoglycan (LPG). This surface molecule was found to target the phagosome membrane where it precluded the recruitment of the acidifying vesicular proton-ATPase (23) and the correct recruitment of protein kinase C (PKC) α (24). Other PKC isoforms, like PKCβ and PKCδ, also showed a disturbed activation during *Leishmania* infections (25–27). Like for PKCα, LPG impaired the proper activation of PKCβ resulting in an inhibition of ROS production (28). This important leishmanicidal mechanism of macrophages was also described as a target of another, intensely studied *Leishmania* virulence factor, the metalloprotease GP63 (also termed leishmanolysin or *Leishmania major* surface protease). The activity of PKC was attenuated by the proteolytic effect of GP63 leading to a diminished release of ROS (29). In this context, the role of mitogen-activated protein kinases (MAPKs), including p38, Jun N-terminal kinase (JNK), and extracellular signal-related kinases (ERK1/2), has been investigated by several groups since they represent major players of the immune pathways present in myeloid cells. In early studies, an absence of MAPK activation during *Leishmania* infection was noted, which was a plausible explanation for the observed hypo-responsiveness of macrophages (30, 31). The ability of *Leishmania* to hinder ERK and p38 phosphorylation was linked to the inability of IFN-γ to induce TNF production in these cells (32). Likewise, the suppression of another proinflammatory cytokine, IL-12, by *L. major* might result from the inhibition of the PKCδ kinase. Mice deficient for PKCδ kinase were more sensitive to *L. major* infection than wild-type mice because of a reduced IL-12p40 and IL-12p70 release by macrophages and dendritic cells and an ensuing Th2-like immune response (33).

Protein kinases are not the only targets of *Leishmania* during infection. Inhibition of IL-12 production by activation of the phosphoinositide 3-kinase (PI3K) during *Leishmania* infection has been convincingly demonstrated using PI3K-deficient mice. These mice developed an improved Th1 response protecting them against *L. major* infection (34). Similarly, activation of the protein kinase RNA-activated (PKR) was important for the intracellular growth of *L. amazonensis* as it promoted the expression of anti-inflammatory cytokine IL-10 and subsequent deactivation of macrophages (35). Interestingly, PKR activation was not observed for all *Leishmania* species. In the case of *L. major*-infected macrophages, PKR activation was actively suppressed by the serine peptidase inhibitor ISP2 of the parasite; activation of PKR by exogenous poly I:C resulted in rapid parasite death due to the release of proinflammatory cytokines (36). Nevertheless, the disease-promoting role of IL-10 in both cutaneous and visceral leishmaniasis has been clearly documented (37–41). Similar to IL-12, its regulation depends on diverse pathways controlled by reversible phosphorylation events. PI3K and the serine/threonine-specific protein kinase AKT (also known as protein kinase B) were shown to be activated during macrophages infection by *L. donovani*. These kinase activities resulted in the inactivation of the glycogen synthase kinase-3β leading to the binding of an activating transcription factor, the cyclic AMP-responsive element-binding protein, to the IL-10 promoter (42).

Not only the production but also the response to proinflammatory cytokines is altered during *Leishmania* infections. JAK2, a tyrosine kinase binding to the receptor of IFN-γ, the key cytokine of protective Th1 cells, was deactivated by a host phosphatase following *Leishmania* infection (43, 44).

**Figure 1:**
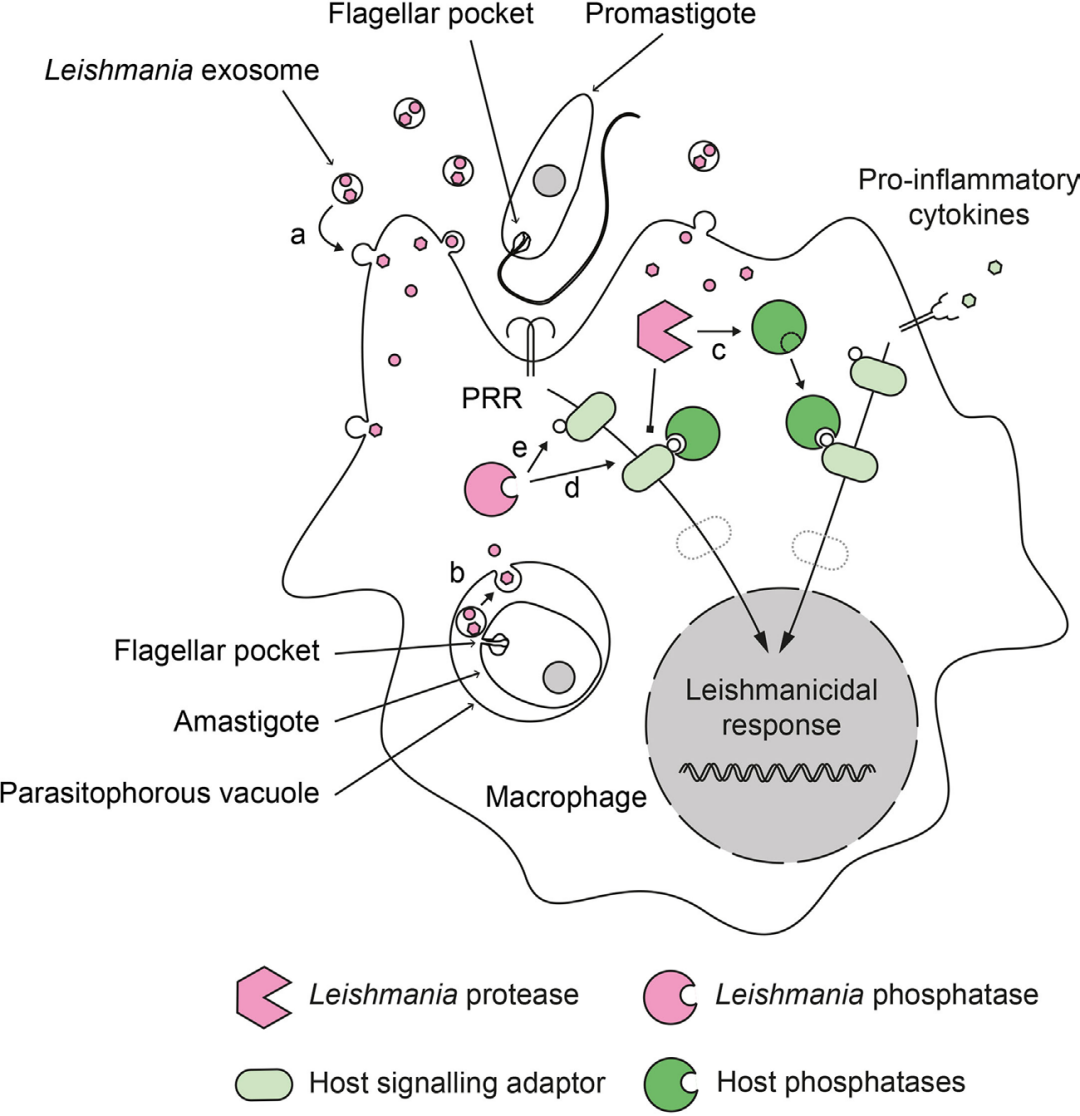
Role of phosphatases in the deactivation of host cell during *Leishmania* infection. *Leishmania* parasites have developed a dual strategy to inactivate important signaling pathways in their host cell. During infection, promastigotes and amastigotes secrete exosomes containing virulence factors. These exosomes release their content into the infected cells after fusing with (a) cytoplasmic membranes (in the case of extracellular promastigotes) or (b) phagosomal membranes (in the case of intracellular amastigotes). Secreted *Leishmania* proteases can proteolytically activate host phosphatases (c) that subsequently down regulate activating cellular pathways. Secreted *Leishmania* phosphatases could synergize with host phosphatases (d) or act independently (e) to modulate the infected cell response.

Altogether, these few examples illustrate the delicate balance between tightly controlled phosphorylation events in the host cells, which are necessary for initiating pathogen control mechanisms, and the ability of the parasite to subvert these reactions. Biochemically, the host-pathogen interaction is governed by kinases (which catalyze the phosphorylation) and by phosphatases, which hydrolyze phosphoester bounds. In the section above, we have briefly mentioned important host kinases involved in the interplay between infected cells and *Leishmania* parasites. In the following part of this review, the role of the phosphatases will be presented. We will focus not only on host phosphatases and how they are modulated by the parasite (e.g., by parasite proteases) but also on *Leishmania* phosphatases and their putative interference with immune cells functions (Figure 1).

## Host Phosphatases

**Figure 2:**
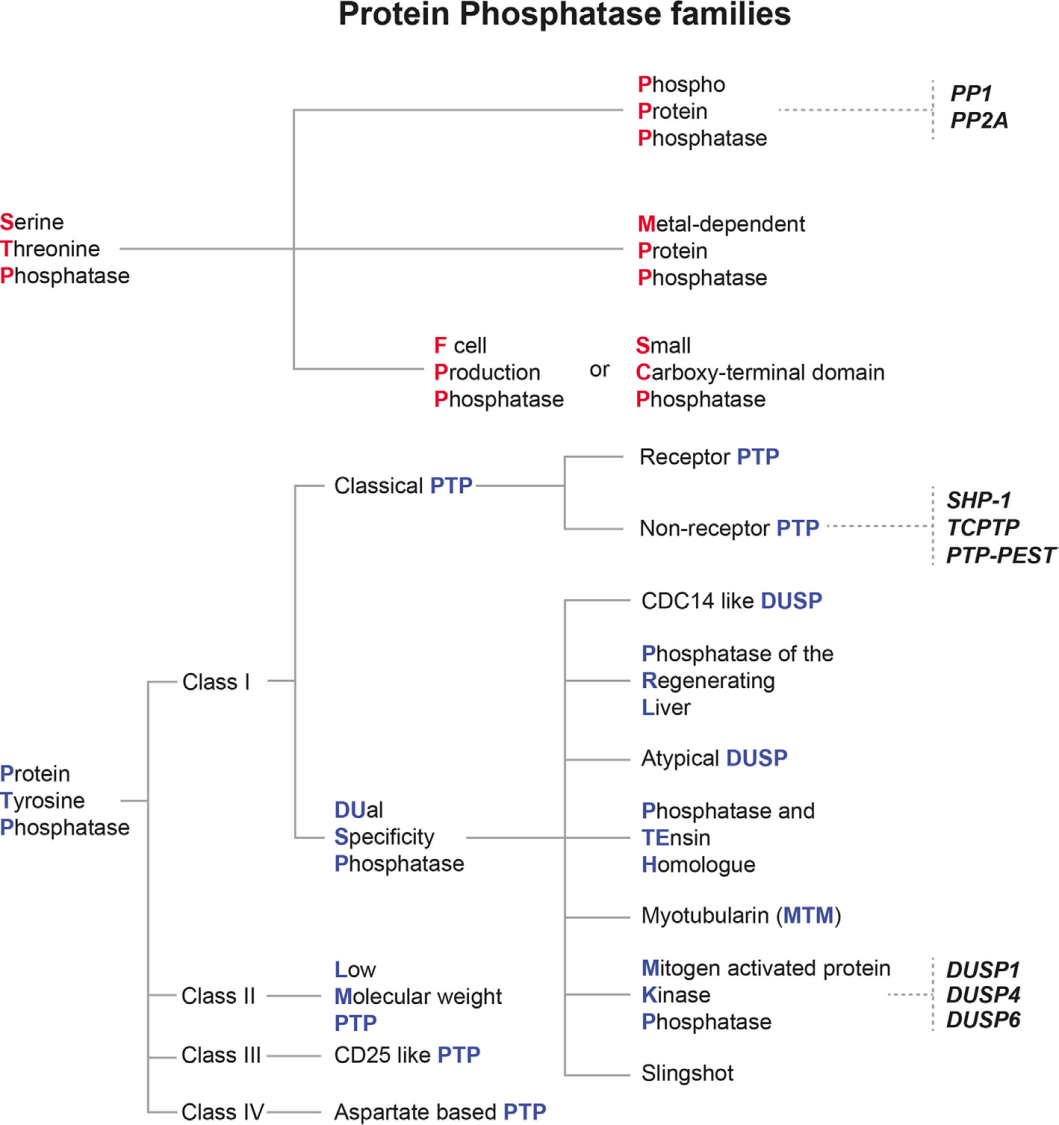
Classification of mammalian protein phosphatase families and their relevance for *Leishmania* infection. Protein phosphatases are classified depending on their structural features and their substrate specificity. The two main families of protein phosphatases and their respective subfamilies are depicted here. Serine threonine phosphatases are marked with red initials, whereas protein tyrosine phosphatases are given with blue initials. Host protein phosphatases which were already shown to affect the outcome of *Leishmania* infections are written in bold and italic black letters.

Protein phosphatases can be classified into two main families of enzymes depending on substrate specificity and catalytic signature. The serine threonine phosphatase (STP) family comprises three main groups: the phosphoprotein phosphatases (PPPs), the metal-dependent protein phosphatases (PPMs), and the aspartate-based phosphatases (F cell production phosphatase [FCP]/small carboxy-terminal domain phosphatase [SCP]) (45). The second family of phosphatases contains enzymes that are able to dephosphorylate phosphotyrosine residues (phosphotyrosine phosphatases [PTPs]), which are also categorized in four different groups depending on their modular organization and their substrate restriction (46) (see Figure 2).

### Serine-Threonine Host Phosphatases Regulating the Outcome of *Leishmania* Infections

Immune cells responding to *Leishmania* infection rely on efficient signaling pathways to stimulate the production of leishmanicidal molecules or to initiate and support their migration, proliferation, and cytokine production. In general, these pathways are finely balanced between an activating signal transmitted by protein kinases and a stopping signal mediated by phosphatases. From the parasite’s perspective, this balance needs to be shifted toward the phosphatase side to guarantee intracellular survival in host cells. Activation of host phosphatases following *Leishmania* infection has been described in macrophages (43) and dendritic cells (47) and helps to explain the reduced immune response observed during visceral leishmaniasis. In mice infected with *L. donovani*, lymphocytes were unresponsive to strong activating and proliferative stimuli like phorbol 12-myristate 13-acetate (PMA) and ionomycin. This defect could be reversed when PKC and ERK phosphorylation were restored by treating the lymphocytes with okadaic acid, an STP inhibitor specific for the heterotrimeric *STP protein phosphatase 2A (PP2A)* (48, 49). In *L. donovani*-infected macrophages activation of PP2A was triggered by host production of ceramide after parasite uptake which resulted in the dephosphorylation of AKT and the subsequent decrease of TNF secretion (50). In striking contrast to these findings are observations made with saliva from the sand fly vector *Phlebotomus papatasi*, which contains a non-proteinaceous inhibitor of the STPs *protein phosphatase 1 (PP1)* and PP2A. As PP1 and PP2A are required for the induction of the iNOS gene (51), the inhibitor present in the sand fly saliva impeded the production of NO by macrophages (52). Thus, both activation and inhibition of PP1 and PP2A can impair the defense against *Leishmania* parasites.

The mitochondrial phosphatase, *phosphoglycerate mutase 5* (*PGAM5*), which was recently described as an atypical, histidine-based STP (53), is another example for a host-protective phosphatase. PGAM5, which acts downstream of receptor-interacting serine/threonine-protein kinase 1 (RIPK1), inhibited the intracellular replication of *Leishmania* (*L. infantum, L. major, L. amazonensis*) in macrophages, presumably *via* induction of IL-1β and NO release. Mice deficient for PGAM5 showed a slight, but significant increase of the parasite load in the skin lesions of *L. amazonensis*-infected mice (54).

### Phosphotyrosine Host Phosphatases Regulating the Outcome of *Leishmania* Infections

A first hint that replication and survival of *Leishmania* in phagocytes is dependent on this family of phosphatases came from the use of inhibitors. *In vitro*, macrophages treated with a PTP inhibitor, the peroxovanadium compound bpV(phen), showed an improved control of *L. donovani* infection, mediated by an increased production of NO (55). Inhibition of ERK2 by a still unknown phosphatase was suggested to account for a similar phenotype seen in a *L. major*-infected human macrophage-like cell line (56). Treatment of macrophages with a related PTP inhibitor, sodium orthovanadate, enhanced the phagocytosis of *Leishmania* promastigotes and thereby allowed a safe entry of the parasite into its cellular niche (57, 58). These *in vitro* results were corroborated by *in vivo* experiments with *L. major*-infected BALB/c mice, in which the PTP inhibitors bpV(phen) and pbV(pic) led to partial parasite control *via* induction of iNOS activity (59). Thus, the activities of PTPs seemed to support intracellular parasite growth.

#### Dual-Specificity Phosphatases (DUSPs)

The therapeutic effect of PTP inhibitor treatments was in line with observations made during *in vivo* treatment of *L. donovani*-infected mice with okadaic acid, a STP PP2A inhibitor (see above). The comparative analysis of both inhibitors in macrophages revealed that PP2A synergistically inhibited the activity of the MAPK ERK1/2 together with a phosphatase belonging to the PTP family, the DUSP6, also known as MAP kinase phosphatase (MKP) 3 (60). The inhibition of MAPKs downstream of PKCζ following *Leishmania* infection led to a decreased iNOS expression (60). In the same study, the role of DUSP1, also termed MKP1, was analyzed. Activation of DUSP1 downstream of PKCε resulted in the inhibition of p38 MAPK (60). A host-damaging role for these two DUSPs during visceral leishmaniasis was also demonstrated in *L. donovani*-infected BALB/c mice treated with 18β-glycyrrhetinic acid (GRA), a compound derived from the medicinal plant licorice (*Glycyrrhizza glabra*). Treatment with GRA caused a shift of the immune response from a detrimental Th2 response to a protective Th1 response (61). At the molecular level, the shift was explained by a decreased DUSP1 and DUSP6 expression and the concomitant activation of mitogen- and stress-activated protein kinase 1 (MSK1), which ultimately led to the production of leishmanicidal NO (62).

Findings with *L. major*-infected macrophages, however, indicated that the activity of DUSP6 is not always detrimental for the outcome of *Leishmania* infections. In *L. major*-infected macrophages activated by anti-CD40, siRNA-mediated inhibition of DUSP6 led to ERK activation, enhanced production of IL-10, and reduced expression of IL-12 and iNOS, whereas inhibition of DUSP1 caused p38 activation and increased generation of IL-12 and iNOS. Consequently, *in vivo* overexpression of DUSP6 or pharmacological inhibition of DUSP1 both ameliorated the course a cutaneous *L. major* infection (63). This functional dichotomy between DUSP6 and DUSP1 has also been reported in the context of macrophage coinfections with the immunomodulatory bacterium *Mycobacterium indicus pranii* and *L. donovani*. The protective effect of this bacterial infection relied on a toll-like receptor (TLR) 4-dependent increase of DUSP6 and simultaneous decrease of DUSP1. Whereas DUSP1 inhibition led to the activation of p38, the production of NO and the release of IL-12, the activation of DUSP6 blocked the ERK1/2 pathway and the expression of IL-10 and arginase-1 (Arg1). Overall, *M. indicus pranii* conferred protection against visceral leishmaniasis by reciprocal regulation of DUSP1 and DUSP6 and a shift toward a Th1 response (64).

A phenotype similar to the one of DUSP6 was also observed for DUSP4 (syn. MKP2). Mice deficient for the *dusp4* gene were more susceptible to a cutaneous infection with *L. mexicana* than wild type mice. This was due to an enhanced JNK and p38 phosphorylation in macrophages that increased the expression of Arg1 and was associated with a disease-promoting Th2 immune response (65, 66).

#### Src Homology Region 2 Domain-Containing Phosphatase-1 (SHP-1)

In macrophages infected with *L. donovani*, a PTP activity identified as *Src homology region 2 domain-containing phosphatase-1* (*SHP-1;* also known as protein tyrosine phosphatase non-receptor type 6 [PTPN6]) was rapidly activated and induced hypo-responsiveness to IFN-γ by directly interacting with the kinase JAK2 recruited to the IFN-γ receptor (31, 43, 44). The confirmation that SHP-1 played a key role in the pathogenicity of leishmaniasis came from infection experiments with viable motheaten (me^v^) mice that are deficient for this phosphatase (67). Me^v^ mice were largely protected against *L. major* infection, most likely because of an increased NO production by their phagocytes (68) and an enhanced secretion of proinflammatory cytokines like TNF, IL-1β, and IL-6 (69). Another group, however, did not observe an impact of the SHP-1 deficiency in me^v^ mice on the course of *L. major*-infection and questioned a selective role of SHP-1 in macrophages as SHP-1 is also regulating T and B cell responses (70). It is also important to point out that even in the absence of SHP-1 activity different species of *Leishmania* (*L. major, L. braziliensis, L. mexicana*, and *L. donovani*) caused degradation and reduced nuclear translocation of STAT1 in primary mouse macrophages (71). This finding indicates that at least part of the suppression of the JAK2/STAT1 pathway in *Leishmania*-infected macrophages and the subsequent reduced release of cytokines and NO in response to IFN-γ is SHP-1 independent.

Infection of SHP-1-deficient macrophages also revealed the importance of this phosphatase for the downregulation of MAPK activity after IFN-γ (31, 44). Control of MAPK by SHP-1 was proposed to be partially responsible for the inhibition of iNOS during IFN-γ stimulation (44, 72). However, involvement of individual MAPK seemed to differ depending on the stimuli used to trigger iNOS expression during *Leishmania* infection. Although SHP-1 negatively regulated ERK1/2 and JNK after IFN-γ stimulation (72), this phosphatase boosted the activity of ERK1/2 after CD40 stimulation, which promoted the secretion of IL-10 and simultaneously inhibited p38 MAPK activation (73).

During *Leishmania* infection, SHP-1 did not only alter the macrophage responses to proinflammatory stimuli originating from T cell such as IFN-γ and CD40 ligand. Signaling pathways triggered by pathogen recognition receptors (PRRs), e.g., TLR4, were also disturbed. In mouse bone marrow-derived macrophages, infection with different *Leishmania* species (*L. major, L. mexicana, L. donovani*) caused rapid activation of SHP-1, which bound to a kinase tyrosyl-based inhibitory motif (KTIM) of the interleukin 1 receptor-associated kinase 1 (IRAK-1) and thereby blocked the intrinsic kinase activity of IRAK-1. The inactivation of IRAK-1 by SHP-1 was associated with the inability of IRAK-1 to detach from the TLR4 adaptor MyD88 and to attach to the downstream signal transducer TRAF6 so that the LPS-induced macrophage responses (e.g., release of NO and TNF) were severely impaired (74). According to the authors of this study KTIMs are also present in other targets of SHP-1, such as ERK1/2, p38, and JNK, and therefore offer a molecular explanation for the multiple effects of SHP-1 (74). A SHP-1 mediated hypo-responsiveness to LPS was also observed in human macrophages infected by *L. donovani* (75).

Two recent studies demonstrated that SHP-1 also modulates the signaling downstream of PRRs of the lectin receptor family during leishmaniasis. C-type lectin receptor Mincle expressed by dendritic cells recognized a still unknown ligand present on the surface of *L. major*. Ligation of the Mincle receptor by *L. major* impaired the activation of dendritic cells due to recruitment of SHP-1, which converted the canonical immunoreceptor tyrosine-based activation motif (ITAM) of the FcRγ-chain signaling component of the receptor into an inhibitory motif (ITAMi). In accordance with these *in vitro* findings Mincle-deficient mice developed less severe skin lesion after infection with *L. major* (76). Likewise, I-type lectin receptors Siglec-1 and -5 expressed by macrophages were described to recognize sialylated ligands on the surface of *L. donovani* parasites. Engagement of Siglec-1 facilitated the uptake of the parasites by the macrophages, whereas ligation of Siglec-5 led to a reduced production of leishmanicidal ROS and NO accompanied by a shift toward a Th2 response. The latter resulted from the recruitment of SHP-1 to the Siglec-5 receptor promoting here again the deactivation of the immune cell (77).

Together, these data illustrate that phosphatases are critical regulators of the immune response against *Leishmania* parasites. Parasite-driven activation of PTPs, notably SHP-1, clearly impairs the effector function of macrophages [reviewed in Ref. (78)]. On the other hand, host cells have developed strategies to counteract the evasion mechanisms of *Leishmania* parasites. As an example, natural resistance-associated macrophage protein 1 (NRAMP-1) inhibited the function of PTPs, either *via* a direct interaction between PTPs and the metal cation (Fe^2+^) transported by NRAMP-1 or by oxidation of the phosphatases resulting from NRAMP-1/iron-dependent ROS-formation (79). However, in the arm race between host and parasite, *Leishmania* parasites were shown to again antagonize this ROS-mediated inhibition of host PTPs in macrophages by inducing the expression of the uncoupling protein 2 (UCP2), a negative regulator of mitochondrial ROS generation. This led to lower ROS production resulting in an enhanced host PTP activity (80).

### Evidence for the Activation of Host Phosphatases by *Leishmania* Virulence Factors

As summarized above, infection of macrophages with *Leishmania* leads to the induction of phosphatase activities, especially SHP-1, which help the parasite to create a safe niche for its replication. Phosphatase activation can be the outcome of normal cell signaling pathways triggered by cytokines and/or PRRs. Alternatively it is possible that microbial virulence factors released inside the host cell directly stimulate phosphatases such as SHP-1. The first evidence that *Leishmania* could do so came from pull-down assays with *L. donovani* extracts and recombinant SHP-1. In these experiments, the *Leishmania elongation factor 1α* (*EF-1α*) was identified as an interactor of the host phosphatase. *Leishmania* EF-1α secreted by intraphago(lyso)somal parasites reached the cytosol of the infected macrophages. Purified *Leishmania* EF1α directly bound to SHP-1 and induced its activity (81), whereas mammalian EF-1α was unable to do so, presumably because of an additional hairpin loop at its surface (82). The secretion of EF-1α by intracellular *Leishmania* amastigotes during macrophage infection was later confirmed and attributed to the exosomes released by the parasites (83).

Production of exosomes by *Leishmania* appears to be the main secretory pathway used by the parasite to export its virulence factors inside and outside the host cells (83, 84). The important contribution of exosomes to the pathogenicity of *Leishmania* was supported (a) by their increased release after a temperature shift mimicking the transit from the sand fly vector (26°C) to the human host (37°C) (85) and (b) by their immunomodulatory effects on innate immune cells (86, 87).

In addition to EF-1α, other proteins contained in *Leishmania* exosomes (83) were found to activate SHP-1. The *fructose-1,6-biphosphate aldolase* was also secreted by *L. donovani* during macrophage infection and could directly interact and activate *in vitro* the phosphatase activity of SHP-1 (88). Unfortunately, the molecular mechanism of SHP-1 activation by both virulence factors, EF-1α and the aldolase, is still unknown.

**Figure 3:**
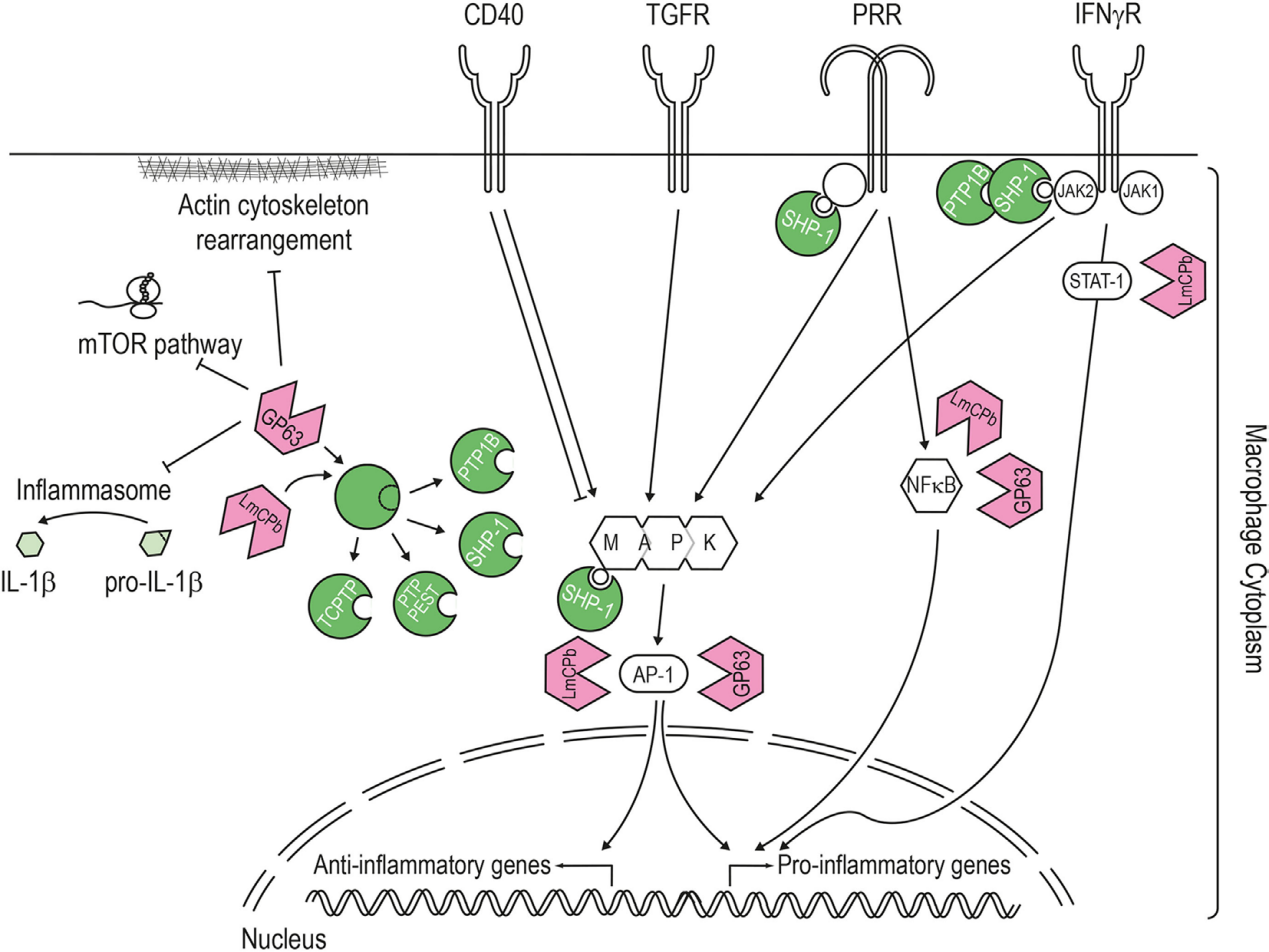
Pathways targeted by the *Leishmania* virulence factors. LmCPb and GP63 proteases are *Leishmania* virulence factors secreted *via* the exosome route and found inside the host cell cytoplasm where they can alter numerous signaling pathways. The best studied *Leishmania* protease is GP63, which proteolytically cleaves and activates host phosphatases (such as PTP1B, PTP-PEST, TCPTP, and SHP-1) or the NF-κB subunit p65/RelA. Once activated, SHP-1 downregulates the macrophage response to interferon-γ by interacting with the kinase JAK2, modulates the pathways induced by pathogen recognition receptors (e.g., toll-like and lectin receptor) and also targets signaling kinases such as mitogen-activated protein kinase. In addition, *Leishmania* proteases contribute to this general downregulation of the host cell response by degrading proteins of the actin cytoskeleton, mTOR (leading to repression of host cell translation), transcription factors (like STAT1 or AP-1), and components of the NLPR3 inflammasome (thereby blocking the generation of IL-1β).

One of the best studied virulence factors of *Leishmania* is the zinc *metalloprotease GP63*. This multifunctional virulence factor is the most abundant C-terminal glycosyl-phosphatidyl-inositol-anchored protein associated with the cell surface of *Leishmania* promastigotes, but is found in limited quantities also inside the parasite (89). On the surface of *Leishmania* parasites, GP63 increased the resistance of promastigotes to complement-mediated lysis by rapidly converting C3b into the inactive complement fragment iC3b (90). iC3b opsonized the parasite and facilitated its silent phagocytosis *via* the CR3 receptor (CD11b/CD18), which synergized with the fibronectin receptor that directly binds GP63 (91). Importantly, GP63, like EF-1α and the aldolase, was also secreted by promastigotes inside exosomes (83, 92). In the cytosol of infected cells, multiple targets of GP63 protease activity have been identified. These include (a) the cytoskeleton adaptors p130Cas (93), cortactin (93) and MARCKS-related protein (94), all of which are degraded by GP63; (b) the translational regulator mTOR (95), which is cleaved by GP63 leading to repression of macrophage translation; (c) PKC, which is also cleaved by GP63 leading to the attenuation of PKC activity (29); (d) transcription factors c-Jun (96) and NF-κB p65/RelA fragment (97), which are inactivated or activated by GP63-mediated proteolysis, respectively; and (e) the host phosphatases SHP-1, PTP1B and TCPTP which are proteolytically activated by GP63 (98). As already discussed before, SHP-1 is a key regulator of many different signaling pathways, including the TLR (74, 75), lectin receptor (76, 77), the MAPK (72, 73, 93) and the IFN-γ-triggered JAK/STAT pathways (44, 68) (Figure 3). It is worth noting that SHP-1 was not the only host phosphatase targeted by GP63 and shown to modulate the activity of JAK2. PTP1B was also proteolytically activated by GP63. Interestingly, whereas under resting conditions both SHP-1 and PTP1B were bound as full-length enzymes to JAK2, in *L. major*-infected macrophages the GP63-activated fragment of PTP1B and the uncleaved (i.e., inactive) SHP-1 prevailed, indicating that PTP1B might have a predominant role compared to SHP-1 in the deactivation of JAK2 (98). The importance of PTP1B activation for parasite survival was further underlined by the increased resistance to *L. major* seen in PTP1B-deficient mice (98).

Two further host phosphatases have been described to be proteolytically activated by GP63 during *Leishmania* infection: TCPTP (92, 93, 98) and PTP-PEST (93). However, their functional relevance for the infection has not been tested so far. Moreover, it seemed that the proteolytic activation of these phosphatases by GP63 was species-specific. GP63-mediated proteolysis was only observed after infection with *L. major, L. donovani*, and *L. mexicana*, but not with *L. tarentolae* and *L. braziliensis* (93).

Another protease, the *cysteine proteinase b* of *L. mexicana* (LmCPb) was proposed to synergize with GP63 to cleave PTP1B. This protease seemed to also participate in the degradation of important transcription factors such as NF-κB, AP-1, and STAT-1 (99). Intriguingly, PTP1B activation only occurred during infection of macrophages by promastigotes, but not by amastigotes (99), unlike SHP-1 that is cleaved after infection with either of both parasite stages, and despite the higher expression of LmCPB in amastigote (100). The authors suggested that amastigotes did not need to target PTP1B, whose regulatory functions overlap with SHP-1, because amastigotes were already more adapted to their niche than promastigotes.

## *Leishmania* Phosphatases

The data summarized in the preceding section illustrated that activation of host phosphatases by *Leishmania* is essential for their infection of mammalian organisms. However, *Leishmania* phosphatases might be equally relevant for the development of the parasite and its survival during the infection process. In the following, we will review the *Leishmania* protein phosphatases already characterized in the context of the parasite life cycle. Next, the role of extracellular acid phosphatases (AcPs) of *Leishmania* will be discussed and compared to a newly described secreted *Leishmania* phosphatase which appears to contribute to the virulence of the parasite.

### Phosphatases Involved in the Life Cycle of *Leishmania* Parasites

Recent technical advances in mass spectrometry allowed systematic analysis of protein phosphorylation in multiple biological contexts. In case of *Leishmania*, studies have initially focused on the differentiation of promastigotes into amastigotes, especially because only a few genes are differentially regulated during this adaptive process, making posttranslational modification a good candidate to regulate this transformation (101). Axenic differentiation of *L. donovani* promastigotes into amastigotes revealed the presence of 627 proteins phosphorylated on 1,614 residues with a majority modified on serine (80%) and threonine (19.4%) and only a few on tyrosine (0.4%). Among them, only 27% of the modifications were present in both developmental stages, whereas 35 and 38% of the phosphorylation were restricted to promastigotes or amastigotes, respectively (102). A refined analysis of the kinetic of differentiation suggested that promastigotes sensed an environmental signal triggering a phosphorylation pathway and resulting in *Leishmania* transformation (103). Like for the stress response protein A2 expressed by *Leishmania* species causing VL (11), gene ontology analysis of the phosphoproteome of *Leishmania* pinpointed the critical role of the heat shock response during differentiation (104). Obviously, parasites need to adapt to the temperature increase experienced during the inoculation into the skin. Indeed, experiments using parasites deficient for heat shock protein (Hsp)70/Hsp90-organizing protein (Hop) (also termed stress-inducible protein 1), a protein belonging to a chaperone complex, proved the importance of phosphorylation in the regulation of the heat shock response (105).

The fine tuning of all these adaptive pathways as well as the metabolic equilibrium of *Leishmania* parasites requires a balanced activation of phosphatases and kinases. Bioinformatic analyses identified 199 protein kinases (106) and 88 protein phosphatases (107) in the genome of the *L. major* species Friedlin. Among the kinases, a few have been identified and functionally characterized. Mainly, the role of MAPK was described in different pathways responsible for sensing environmental changes and ranged from flagellar control (108) to arginine deprivation (109). Regarding protein phosphatases, their characterization is still patchy as we will see in the following paragraphs (Tables 1 and 2) [reviewed in Refs. (107, 110)].

**Table 1:** Serine threonine phosphatases of *Leishmania major*.^a^

Family			*L. major* gene	Characterized *Leishmania* gene	Reference
STP	PPP	PP1	LmjF.15.0220		
			LmjF.28.0690		
			LmjF.31.2630 LmjF.34.0780		
			LmjF.34.0790		
			LmjF.34.0800 LmjF.34.0810 LmjF.34.0850		
		
		PP2A	LmjF.25.1320 LmjF.28.2670		
		
		PP2B	LmjF.26.2530 LmjF.36.1980		
		
		PP4	LmjF.32.3040	LdBPK_323230.1	(111)
		
		PP5	LmjF.18.0150		(112)
		
		PP6	LmjF.34.4190		
		
		PP7 (PPEF)	LmjF.12.0660	LinJ.12.0610	(113, 114)
		
		ApaH-like(ALPH)	LmjF.22.1600 LmjF.17.0580		
		
		Shewanella-like	None		
		
		kSTP (pseudo)	LmjF.05.0100		
			LmjF.09.0470 LmjF.12.0050 LmjF.13.1510 LmjF.13.1570 LmjF.22.1490 LmjF.24.0270 LmjF.26.2100		
			LmjF.34.2770	LinJ.34.2610	(115)
			LmjF.36.2050		
			LmjF.29.0440 LmjF.30.3280		
	
	MPP (PP2C)		LmjF.14.0900 LmjF.15.0170 LmjF.36.0530 LmjF.32.1690 LmjF.27.2320 LmjF.36.1230 LmjF.30.0380 LmjF.25.2060 LmjF.06.0900			
			LmjF.25.0750	LinJ.25.0780	(85, 116)
			LmjF.31.1320		
			LmjF.34.2500	LinJ.34.2310	(114)
			LmjF.34.2510	LinJ.34.2320	(114)
			LmjF.27.1180 LmjF.34.0730		
	
	FCP SCP		LmjF.26.0160 LmjF.34.3920 LmjF.32.0100 LmjF.25.2030 LmjF.24.0290 LmjF.34.1250 LmjF.35.2620 LmjF.36.6780 LmjF.35.3520 LmjF.27.2180 LmjF.29.2400 LmjF.35.0190 LmjF.03.0890		

*^a^Phosphatases identified by mass spectrometry in the secretome of *Leishmania* parasites are marked in *red* (83–85, 92)*.

*STP, serine threonine phosphatase; PPP, phosphoprotein phosphatase; MPP, metal-dependent phosphatase; FPP, F cell production phosphatase; SCP, small carboxy-terminal domain phosphatase; PP, protein phosphatase; PPEF, protein phosphatases with a E-helix/F-helix (EF)-hand (PPEF); kSTP, kinetoplastid specific STP*.

**Table 2:** Tyrosine phosphatases of *Leishmania major*.^a^

Family					*L*. major gene	Charact. gene	Ref.
PTP	Class I		Receptor		Nd		
			
		Classical PTP	Non-receptor PTP	Eukaryotic like	LmjF36.5370 LmjF36.2180	LdBPK_365610.1	(117)
				
				Kinetosplastid	LmjF32.0640		
		
			eDUSP PRL		LmjF16.0230		(118)
					LmjF16.0250		
			
			eDUSP CDC14		LmjF09.0420		
			
			Atypical DUSP	LRR-DUSP	LmjF28.0170		
				
				Kinatase	LmjF34.2190		
				
				ANK-DUSP	Nd		
				
				STYX	LmjF21.0700		
				
				MKP-like	LmjF27.1840		
				
				Lipid-like phosphatases	LmjF04.0560 LmjF25.0570 LmjF33.2840		
					LmjF22.0250		(119)
			
		DUSP	PTEN	Eukaryotic-like	LmjF34.1430		
				
				Kinetosplastid	LmjF25.0230		
			
			MTM		LmjF20.1480 LmjF12.0320		
			
			Kinetoplastid DUSP		LmjF03.0510 LmjF05.0220 LmjF08.0100 LmjF09.0550 LmjF13.0770 LmjF27.2210 LmjF28.0790 LmjF35.4650 LmjF32.1010 LmjF04.0840 LmjF05.0720		
			
			MAKP phosphatase		Nd		
			
			Slingshot		Nd		
	
	Class II	LMPTP/ArsC reductase		LmjF01.0200			
	
	Class III	CDC25/ACR2 reductase		LmjF32.2740			(120–123)
	
	Class IV	Asparate based PTP EyA		Nd			

*^a^Phosphatases identified by mass spectrometry in the secretome of *Leishmania* parasites are marked in *red* (83–85, 92)*.

*PTP, protein tyrosine phosphatase; DUSP, dual specificity phosphatase; PRL, phosphatase of the regenerating liver; STYX, inactive STYX pseudo-phosphatase; MKP, mitogen activated protein kinase phosphatase; PTEN, phosphatase and tensin homologs; MTM, myotubularin; LMPTP, low-molecular -weight PTP; ArsC red/ACR2, arsenate reductase*.

#### *Leishmania* Protein Serine/Threonine Phosphatases

*Leishmania major*, the model species used for this review, encodes 58 genes for serine/threonine phosphatases (STP) which are members of the three different groups of enzymes within this family: the PPP group (30 genes), the PPMs (15 genes), and the aspartate-based phosphatases (FCP/SCP; 13 genes) (see Table 1). These enzymes are highly conserved in the different *Leishmania* species (Table S1 in Supplementary Material).

Within the *PPP-group*, the eight *Leishmania* phosphatases that belong to the PP1 subgroup have not yet been biochemically and/or functionally characterized. Surprisingly, five of them are encoded by genes organized in tandem on chromosome 34. This organization suggests a common regulation of the expression of the different isoforms. PP2A phosphatases are also not yet fully characterized, but their increased expression has been linked to resistance against antibiotics such as paromomycin (111). Additional information on their functions in *Leishmania* parasites could be derived from studies of orthologs in other kinetoplastid. PP1 and PP2A phosphatases of *Trypanosoma* parasites participate in amastigote transformation (110) and, interestingly, share the highest homology with *Leishmania* orthologs (Table S1 in Supplementary Material).

Regarding the calcineurin-like phosphatase subgroup (PP2B), a calcium- and calmodulin-dependent phosphatase activity was purified from *L. donovani* parasites but their respective genes have not yet been sequenced (124). In *L. major* two genes, *LmjF26.2530* and *LmjF36.1980*, might account for this activity (Table 1) as they encode proteins carrying a calcineurin A catalytic domain and the three canonical regulatory domains: the calcineurin B (CnB) binding domain, the calmodulin (CaM) binding domain and an autoinhibitory domain (possibly missing in *LmjF36.1980*) (125). Noteworthy, the ortholog found in *T. cruzi* does not contain the last two domains which likely causes a substantial functional difference (125). Interestingly, *Leishmania* CnB (*LmjF.21.1630*), the regulatory subunit of calcineurin A, was shown to control the temperature adaptation of *L. major*. Promastigotes deficient for CnB failed to differentiate into amastigotes and were completely avirulent in macrophages and during *in vivo* infection (126).

The PP7-like phosphatase, encoded by the gene *LmjF.12.0660*, showed a strong homology to protein phosphatases with a E-helix/F-helix (EF)-hand (PPEF) that were identified in sensory cells of higher eukaryotes. However, LmPPEF did not bind calcium because its EF-hand domains appeared degenerated and the functionality of its N-terminal calmodulin binding domain was not ascertained (113). Due to a N-myristoylation modification, LmPPEF appears to be membrane-bound and localized in the vicinity of the kinetoplast and the flagellar pocket (113), an active secretory site, which might explain its detection in the exosome content of promastigotes (83, 92).

PP5 phosphatases showed some similarities with PP7 proteins, but instead of calcium-dependent regulatory domains they carry N-terminal tricopeptide repeat (TPR) domains, which can mediate protein–protein interaction and autoinhibition. The PP5 phosphatase encoded by *L. major* (*LmjF18.0150*) has been biochemically characterized. Mutation analysis of the recombinant protein confirmed its phosphatase activity and the autoinhibitory role of the TPR domains (112). Functional data on this protein are scarce but suggested its involvement in stress response as also seen with the *T. brucei* homolog (110). Expression of the unique PP5 gene in *L. infantum* correlated with increased resistance to antimony (114).

Regarding the eukaryotic-like PP6 phosphatases or the eukaryotic phosphatases homologous to the bacterial enzyme diadenosine tetraphosphate hydrolase ApaH (ApaH-like phosphatases [ALPHs]), only few data exist on their biochemical or physiological role. In *Trypanosoma brucei*, ALPH1 was shown to have all features of an mRNA decapping enzyme (127).

The last subgroup of the PPPs comprises kinetoplastid-specific proteins (kPPP) that carry a mutation in their catalytic site. These pseudo-phosphatases (kSTP pseudo) show some similarities to plant and fungal PPPs. They likely add an extra level of regulation to the kinase/phosphatase activities by stably binding to phosphorylated substrates and shielding them against other phosphatase activities (110). These adaptations to numerous substrates combined with the absent catalytic activity might explain the higher divergence observed between kPPP from *Leishmania* and *Trypanosoma* (Table S1 in Supplementary Material). Expression of such pseudo-enzyme, encoded by the gene *LinJ.34.2610*, has been negatively correlated with the resistance of *L. infantum* to antimony treatment (115).

Regarding the 15 phosphatases of the *PPM (or PP2C) group*, their biological functions have not yet been unraveled, but their dependency on divalent manganese or magnesium cations has been validated in *L. donovani* and *L. infantum/L. chagasi* promastigotes (116, 128). In case of *L. infantum/chagasi*, a 42 kDa PP2C-like phosphatase named LcPP2C, encoded by the gene *LinJ.25.0780*, was biochemically characterized in promastigotes and amastigotes (116). Interestingly, the ortholog of this protein was secreted by *L. mexicana* along with 71 other proteins detected (84, 85). However, whether this enzyme contributes to the modulation of the host cell signaling (e.g., *via* activation of the host phosphatases SHP-1 and PTP-1B) by the *L. mexicana* secretome, was not investigated (85). Other PP2C-like proteins have been detected in *L. mexicana* promastigotes but they still lack functional characterization (129). Moreover, expression of two additional PP2C genes, *LinJ.34.2310* and *LinJ.34.2320*, was recently linked to methotrexate resistance of *L. infantum* (114).

Altogether, only 6 of the 58 STP genes have been partly characterized despite the suggested crucial role in amastigote transformation (Table 1). More research on this phosphatase family could therefore open new avenues toward the development of alternative therapeutic approaches.

#### *Leishmania* Protein Tyrosine Phosphatases

In additions to STPs, 31 putative protein tyrosine phosphatases (PTPs) are encoded in the *L. major* genome (Table 2). All of them share a common catalytic site surrounded by a cysteine and an arginine (CX_5_R). They are well conserved among *Leishmania* species and are divided into four classes (Table S1 in Supplementary Material). *Class I PTPs*, which form the largest group, contain enzymes with either proven PTP activity or a structural organization characteristic for PTPs, whereas classes II and III, represented by a single member each, might not convey such activities. Class IV enzymes were not detected in the genome of *Leishmania* parasites.

Proteins of the *class II PTPs* are homologous to low-molecular-weight (LMW) PTPs found in human or bacteria. However, the protein encoded by the gene *LmjF.01.0200* carries a mutated catalytic cysteine as revealed during our own sequence analyses, suggesting that it is probably inactive and might therefore act, similar to the kPPPs, as substrate trapping molecule. Another degenerated LMW PTP-like enzyme is encoded by the gene *LTRL590_050007600.1*, whose expression correlated to antimony resistance in *L. tropica* field isolates (130).

Regarding the *class III PTPs*, the enzyme named LmACR2 has been initially characterized in *L. major* as an arsenate (As)/antimony (Sb) reductase despite containing a canonical PTP catalytic site. The antimony reductase activity of LmACR2 converted Sb(V), present in Sb-containing antileishmanial drugs (e.g., sodium stibogluconate), into Sb(III) which is the active microbicidal molecule. Overexpression of LmACR2 in promastigotes supported this observation as parasites became more sensitive to antimonials (120). However, metalloids like Sb are not commonly encountered by *Leishmania* parasites in nature. Therefore, the evolutionary pressure to develop such activity should have been low. Thus, the concomitant PTP activity of LmACR2 detected *in vitro* might reflect the true physiological function of this enzyme (121, 122).

Most of the PTPs expressed in *Leishmania* parasites belong to the *class I PTPs* that can be further divided into two subgroups of enzymes. The first subgroup consists of classical PTPs homologous to human PTP1B. However, in *Leishmania* and other kinetoplastid parasites, this group does not contain any receptor-PTP usually found in higher eukaryotes. Among the three non-receptor-PTPs encoded by *Leishmania* parasites, only the eukaryotic-like LdPTP1 was functionally characterized. The deletion of the gene encoding for this enzyme, *LdBPK_365610.1*, resulted in complete loss of amastigote transformation during infection of macrophages with *L. donovani* promastigotes and in a dramatic decrease of the parasite virulence *in vivo* (117). The molecular mechanism underlying this drastic phenotype has not yet been elucidated. However, the study of the TbPTP1 ortholog in *Trypanosoma brucei* revealed that this family of phosphatase could control the integration of environmental differentiation signal (131, 132).

The second subgroup of class I PTPs comprises dual specificity phosphatases (DUSP) (Table 2). These phosphatases are characterized by a substrate spectrum that extends beyond phosphotyrosine residue (e.g., dephosphorylation of serine and threonine residues). Some of these DUSPs are even described as lipid phosphatases:

(a) Myotubularins (MTMs) were shown to target phosphoinositides like PI(3)P or PI(3,5)P_2_ in mammals, and therefore control endocytosis and membrane trafficking (133). In *Leishmania* parasites, the MTM group consists of only two genes (instead of the 16 encoded by the human genome making it the largest group of human DUSP). *Leishmania* MTMs are characterized by their very large size with up to 3,245 amino acid (as in the case of *LmjF.12.0320*), which is due to long N-terminal extensions. However, their function has not been yet studied in trypanosomatids.
(b) Two *Leishmania* genes encode still uncharacterized phosphatase and tensin homologs (PTEN) that are well known to target PI(3,4,5,)P_3_ in mammals. Whereas one gene is homologous to kinetoplastid PTENs, the other one shares homology with eukaryotic phosphoinositide phosphatases. Interestingly, the latter subgroup is not represented in all Trypanosomatidae, as it is missing in *T. brucei* (Table S1 in Supplementary Material).
(c) A group of four *Leishmania* DUSPs encodes lipid-like phosphatases that have similarities with bacterial virulence factors. *LmjF.22.0250* was identified as a close homolog of the secreted PIP phosphatases MptpB from *Mycobacterium tuberculosis* (119) and LipA from *Listeria monocytogenes* (134). Strikingly, *LmjF.22.0250* is the only protein phosphatase gene that is not shared by all *Leishmania* species or strains as it was absent from the genome of *L. panamensis* MHOM/COL/81/L13 and *L. braziliensis* MHOM/BR/75/M2903, but present in *L. braziliensis* MHOM/BR/75/M2904 (Table S1 in Supplementary Material). As this phosphatase was not found to be secreted, the absence of *LmjF.220250* might be compensated by the other three members of this group of lipid-like phosphatases encoded in the genome of *Leishmania*. Nonetheless, lipid phosphatases might contribute to the parasite virulence, because one of them, *LbrM.25.21.80* was among the 100 genes most abundantly expressed by *L. braziliensis* in skin lesions of humans with CL (135).

In addition to these phospholipid phosphatases, DUSPs dephosphorylating proteins on tyrosine, serine or threonine residues were also identified in the *Leishmania* genomes:

(a) Three *Leishmania* genes encode eukaryotic-like DUSPs (eDUSPs). These include a homolog of the human CDC14 phosphatase that was described to control mitosis by targeting cyclin dependent kinase (136). However, this crucial function has not been yet documented in trypanosomatids. The two genes homologous to phosphatase of the regenerating liver (PRL) will be discussed later in the context of their importance for the virulence of *L. major*.
(b) In addition to the lipid-like phosphatases discussed above, the group of atypical DUSPs encodes for (i) a kinetoplastid MAP kinase-like phosphatase (MKP) lacking a functional rhodanese homology domain, (ii) a leucine-rich repeat (LRR) DUSP, (iii) a kinatase having LRR domains and a pseudokinase domain, and (iv) an inactive STYX pseudo-phosphatase. None of these enzymes has yet been characterized in Trypanosomatidae.
(c) The remaining *Leishmania* DUSPs belong to the group of still uncharacterized kinetoplastid-specific DUSPs.

Although the large majority of the *Leishmania* DUSPs have so far not been functionally studied in *Leishmania*, it is interesting to note that two of them (LmjF.16.0230 and LmjF34.2190) were secreted in exosomes by *Leishmania* parasites (83, 92) (Table 2). These two DUSPs along with the six STPs also detected in exosomes (i.e., LmPPEF, LcPP2C, two PP1, and two kSTPs) represent a small pool of *Leishmania* phosphatases that might directly interact with phosphorylation cascades inside host cells during infection.

### *Leishmania* Membrane and Secreted AcPs

Until this year, direct modulation of host responses by *Leishmania* phosphatases had only been documented for AcPs. These phosphatases do not belong to the classical STP or PTP families because of the unique composition of their catalytic site that is based on histidine residues. In the literature, histidine phosphatases are usually not discussed along with other protein phosphatases as their substrate specificity has not been ascertained. AcP activities have been described against various substrates, including glycerophosphate, fructose-1,6-diphosphate (137, 138), phosphoinositides (139), pyrosphosphate (140), and phosphoproteins (138, 141).

**Figure 4:**
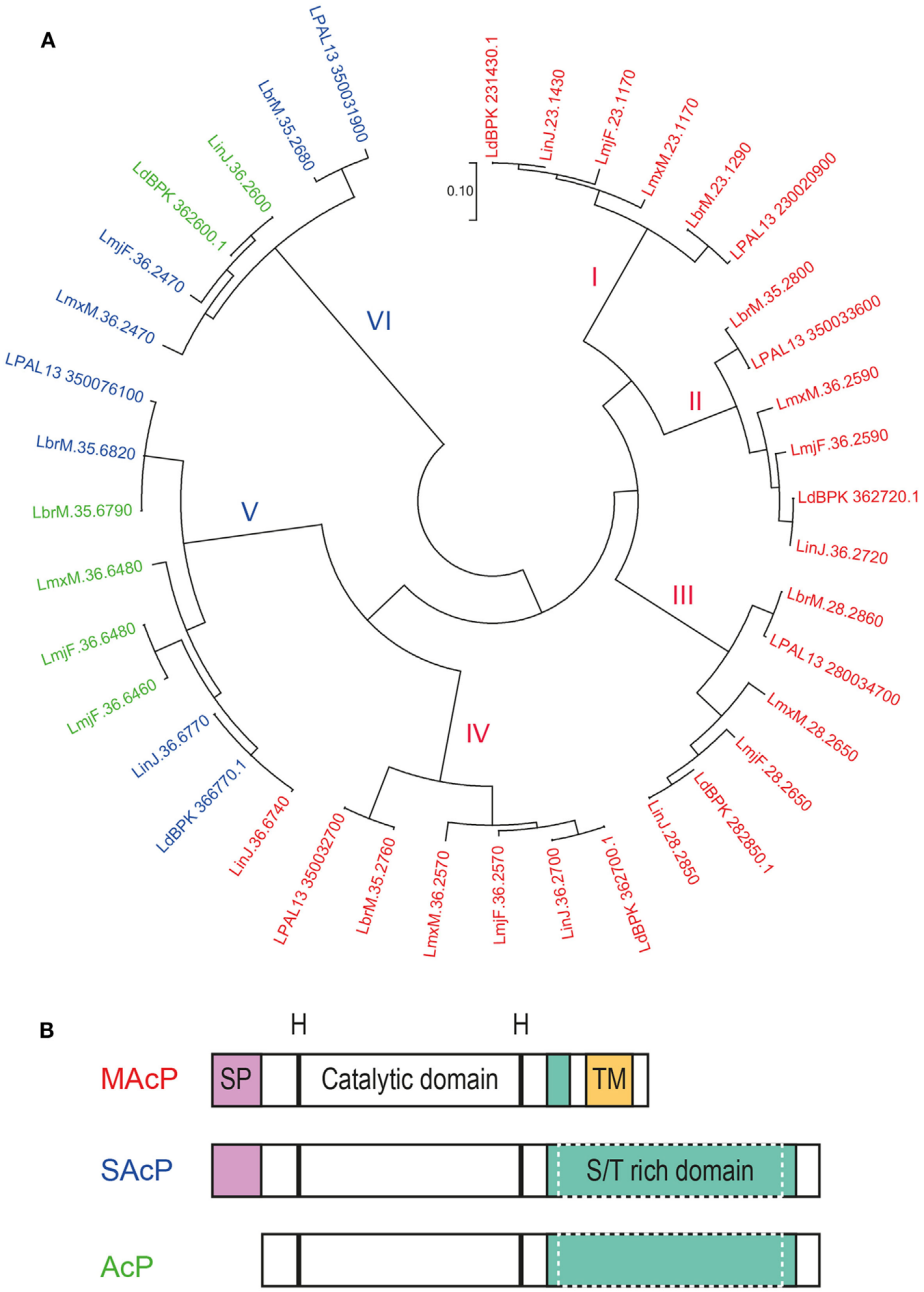
Acid phosphatases (AcPs) of *Leishmania* parasites. AcP, secreted by *Leishmania* parasites or expressed on their surface, belong to the family of histidine phosphatases. **(A)** Phylogenetic comparison between histidine phosphatases of *L. major* Friedlin, *L. infantum* JPCM5, *L. donovani* BPK282A1, *L. mexicana* MHOM/GT/2001/U1103, *L. braziliensis* MHOM/BR/75/M2904, *L. panamensis* MHOM/COL/81/L13. Available gene sequences were extracted from the TriTrypDB Kinetoplastid Genomics Resource database (IPR: 000560) and were used to construct a phylogenetic topology with the minimum evolution method. The evolutionary distance (scale) is measured in number of base substitutions per site. The evolutionary history was inferred using the Minimum Evolution method (142). The evolutionary distances were computed using the Maximum Composite Likelihood method (143) and are in the units of the number of base substitutions per site. The minimum evolution tree was searched using the Close-Neighbor-Interchange (CNI) algorithm (144) at a search level of 1. The Neighbor-Joining algorithm (145) was used to generate the initial tree. The analysis involved 39 nucleotide sequences. All positions containing gaps and missing data were eliminated. There was a total of 409 positions in the final data set. Evolutionary analyses were conducted in MEGA7.0.18 (146). Protein sequences of the respective genes were analyzed for the presence of a putative signal peptide (SP) and transmembrane domain (TM) with the Phobius software (147). Genes are labeled in red for the membrane AcP (MAcP) containing a signal peptide (SP) and transmembrane domain (TM), in blue for the secreted AcP (SAcP) containing only a SP and in green for the phosphatases lacking both domains. **(B)** Schematic organization of the protein domains of *Leishmania* AcP. Membrane and secreted AcP have a SP at their N-terminus followed by a catalytic domain based on histidine residues. Next, AcP harbor a serine/threonine(S/T)-rich domain that varies in length and can be significantly larger in SAcPs. Finally, MAcPs harbor a TM close to their C-terminus. Some AcPs (green in panel A) are devoid of SP and TM but can harbor long S/T-rich domains.

A search for histidine phosphatases in the genome of six species of *Leishmania* pathogenic for humans (*L. major, L. mexicana, L. braziliensis, L. panamensis, L. infantum, L. donovani*) using their signature motif (Interpro accession reference for the histidine phosphatase superfamily: IPR000560) revealed that each *Leishmania* genome contained six or seven genes of this family. Extending our search to pathogenic *Trypanosoma* species (*T. brucei* and *T. cruzi*) revealed that this family of phosphatase is well conserved among the Trypanosomatidae. These genes are distributed in two main groups with four to five genes encoding membrane acid phosphatase (MAcP; also known as ectophosphatases) and two to three genes encoding secreted acid phosphatases (SAcP) (Figure 4A; Table S2 in Supplementary Material).

#### Membrane Acid Phosphatases

All MAcPs share the same structural organization starting with a signal peptide at the N-terminus followed by the catalytic domain containing the histidine residues and a transmembrane domain at the C-terminus (Figure 4B). Phylogenetically, MAcPs are organized in four distinct groups (Figure 4A, groups I–IV), all represented by one MAcP gene sequence derived from each of the six analyzed *Leishmania* genomes. Interestingly, an additional MAcP gene, *LinJ.36.6740*, could be detected in the genome of *L. infantum*. Despite being more related to SAcP and being substantially shorter than all other MAcP (only 35 kDa instead of usually around 60 kDa), this phosphatase harbors a signal peptide and a putative transmembrane domain at its end, illustrating the genetic plasticity of *Leishmania* parasites in which diverging paralogous genes are common. Gene divergence was also seen in the MAcP gene of *L. donovani*. The MAcP protein of the *L. donovani* strain MHOM/SD/62/1S-CL2D (148) carried a transmembrane domain, which was found to be replaced by a not-functionally defined C terminal sequence of 91 amino acids in the MAcP gene of the *L. donovani* strain BPK282A1 (149). Comparing the published sequences, the variation between these *Leishmania* strains was not related to frameshifts or aberrant stop codons but might result from a more complex evolutionary process.

Numerous studies aimed to characterize the function of MAcPs as they represented the first enzymatic activities identified on the surface of *Leishmania* promastigotes (150). Having been transported *via* the classical intracellular secretion pathway, it was an expected finding that the MAcPs were phospho-glycosylated (151). Interestingly, expression of MAcP varied during the different life stages of *Leishmania* parasites. In *L. major*, MAcP expression increased during metacyclogenesis of promastigotes and correlated with the translocation of the protein from the cytoplasm to the membrane (152). Increased expression of some of the MAcPs was also detected during metacyclogenesis and differentiation into amastigotes following macrophage infection (153, 154). Most recently, MAcPs were found to be differentially expressed in clinical isolates of the same *Leishmania* species (155). A MAcP of *L. tropica* (LTRL590_280034100, annotated as LmjF.28.2650) was described as one of the 30 most diversely expressed genes in a collection of 14 clinical isolates of *L. tropica* from different geographic areas. This might indicate that the parasite adapts the level of extracellular phosphatase activity depending on the specific infection situation. Functionally, early reports suggested that these ectophosphatases altered the response of neutrophils (156). Partially purified MAcP activity from *L. donovani* specifically interfered with the production of ROS by neutrophils after stimulation with the tripeptide *N*-formylmethionine-leucyl-phenylalanine (fMLP), whereas activation by PMA was resistant to MAcP activity (157). Other authors proposed that a phosphoinositide phosphatase activity of MAcP might play a role in this inhibition, because the phagocyte NADPH oxidase (Phox) relies on these signaling molecules for its activation (139). In accordance with this hypothesis, overexpression of LdMAcP by *L. donovani* promastigotes resulted in an increased survival during *in vitro* infection of a mouse macrophage cell line (158). However, deletion of the MAcP gene of *L. mexicana* (*LmxM.36.2570* [LmxMBAP]) raised doubts about an essential role of this class of phosphatases for the virulence of *L. mexicana* parasites; LmxMBAP-deficient parasites were as virulent as wild-type parasites during *in vitro* infection of macrophages or *in vivo* infection of susceptible BALB/c mice (159). However, a definitive statement on the function of MAcPs is still not possible because of potential compensatory mechanisms as *Leishmania* parasites carry a minimum of four MAcP genes.

#### Secreted Acid Phosphatases

Secreted acid phosphatases are among the most abundant proteins secreted by *Leishmania* parasites (137, 160). However, a few *Leishmania* species, such as *L. major*, were shown to express very little SAcP as promastigotes (161). This difference could be due to the number of genes coding for truly secreted phosphatases. Our *in silico* analysis of the three *L. major* histidine phosphatases devoid of transmembrane regions revealed that only one, *LmjF.36.2470*, might have a functional signal peptide (Table S2 in Supplementary Material), whereas other *Leishmania* species such as *L. panamensis, L. braziliensis, L. mexicana*, and *L. donovani* can express more than one intact SAcP. The phylogenetic analysis also allowed the classification of SAcPs in two different families that are substantially divergent. One group of phosphatases (Figure 4A, group VI) branched out early from the histidine phosphatase family but is highly conserved across species, while the other group (Figure 4A, group V) is more closely related to MAcPs but is also more diverse. In members of group V, a signal peptide is sometimes absent, the enzymes can be significantly shorter than other orthologs and truncation could occur in the catalytic domain. This is the case for the two putatively non-secreted phosphatases of *L. major, LmjF.36.6460*, and *LmjF.36.6480*, indicating that the function of these two genes probably differs from their orthologs in case they are expressed.

For parasites expressing large amounts of SAcP, such as *L. donovani*, soluble phosphatase activity could be detected all along their differentiation from promastigotes to amastigotes (162). Interestingly, SAcP activity was also found in the cytosol of macrophages after 24 h of infection (11). However, the exact molecular identity of the phosphatase was not determined and this family of enzymes was also not detected inside exosomes that represent the main transit route of *Leishmania* cargo toward host cell cytosol. Like MAcPs, SAcPs are phospho-glycosylated proteins (163, 164). This modification relies on the same family of enzymes responsible for the LPG biosynthesis as a deletion of *lpg2* gene, but not *lpg1*, abolished the phospho-glycosylation of SAcPs in *L. donovani* (165). Immunoprecipitation of SAcP suggested that the glycosylation pattern might increase during the parasite differentiation into amastigotes (11, 162). In addition to this posttranslational modification, SAcPs can form filamentous polymers. This quaternary structure has been extensively described for *L. mexicana*, but, surprisingly, was not seen with SAcPs from *L. donovani*, which remained mono- or oligomeric and non-filamentous (166). In case of *L. mexicana*, the filament formation occurred in the flagellar pocket of promastigotes (167) to create homo- or hetero-polymers of two phosphatases, LmSAP1 and LmSAP2. The genes encoding these two SAcPs are tandemly arranged and nearly identical, only differing by the addition of a long stretch of serine-threonine repetitions after the catalytic domain of LmSAP2 (168). This stretch is the site of intense glycosylation but does not interfere with the formation of the filament backbone constructed around the catalytic domains of LmSAPs (169). Remarkably, the published sequences of LmSAPs derived from *L. mexicana* MNYC/BZ/62/M379 could not be fully retrieved from the *L. mexicana* MHOM/GT/2001/U1103 strain that was sequenced, illustrating again the high plasticity of *Leishmania* genomes. An ortholog of the LmSAPs, *LmxM.36.6480*, has even a higher number of serine-threonine repetitions and seems to lack a functional signal peptide. In general, our search of the different *Leishmania* genomes showed that all SAcPs share a similar architecture but the size of the serine/threonine rich domain varies. This probably reflects the need to adapt the level of phospho-glycosylation to the different environments required by each parasite species.

Comparative genetic analysis of two different isolates of *L. major* from patients with contrasting severity of disease indicated that the hypo-virulent strain carried a mutated version of SAcP (170), suggesting a role of SAcP in virulence. Interestingly, a similar study looking at the genetic differences between two strains of *L. donovani* isolated from patients with cutaneous or visceral leishmaniasis, respectively, did not report a link between *Leishmania* AcPs and virulence (171). The function of SAcP in the virulence *Leishmania* has been directly addressed with parasites lacking the genomic locus encoding the two LmSAPs. Deletion of this locus abolished the virulence of *L. mexicana* promastigotes as they failed to transform into amastigotes during macrophage infection (172). However, this phenotype was independent of the LmSAPs because phenotypic complementation was achieved by adding back the MAPK gene that was identified in the intergenic region between the two LmSAP genes (172).

Together, these results demonstrate that the exact contribution of MAcPs and SAcPs to maintain the life cycle of *Leishmania* parasites is still unknown. First, the lack of a phenotype in a *Leishmania* mutant deficient for an AcP does not exclude its functional relevance, because compensation between different genes of each group could occur. Second, MAcP could also compensate for SAcP deficiency and *vice versa*. Finally, a role for these phosphatases also needs to be considered during sand fly infection by promastigotes. To experimentally address these hypotheses, the nature of the AcP substrate needs to be defined. If, however, these enzymes are primarily responsible for providing sufficient inorganic phosphate to the parasite as it has been proposed by Freitas-Mesquita et al. (140), it will be difficult to dissect the functional role of the different classes of AcPs and/or to overcome compensatory mechanisms.

### *Leishmania* Phosphatase LmPRL-1 As a Secreted Virulence Factor

Recently, our laboratory has characterized a new dual specificity phosphatase, LmPRL-1, secreted by *L. major* during macrophage infection (118). The LmPRL-1 gene, *LmjF.16.0230*, is organized in tandem with a paralogous gene, *LmjF.16.0250*, which codes for LmPRL-2. Both PTPs represent the only two members of the family of the *phosphatase of the regenerating liver* (*PRL*) within *L. major* (Table 2). Human PRLs are well studied and are thought to participate in the control of various processes such as cell proliferation, differentiation and motility (173). Remarkably, both LmPRLs are highly conserved in all sequenced *Leishmania* species indicating that they might play a crucial role in the life of the parasite.

**Figure 5:**
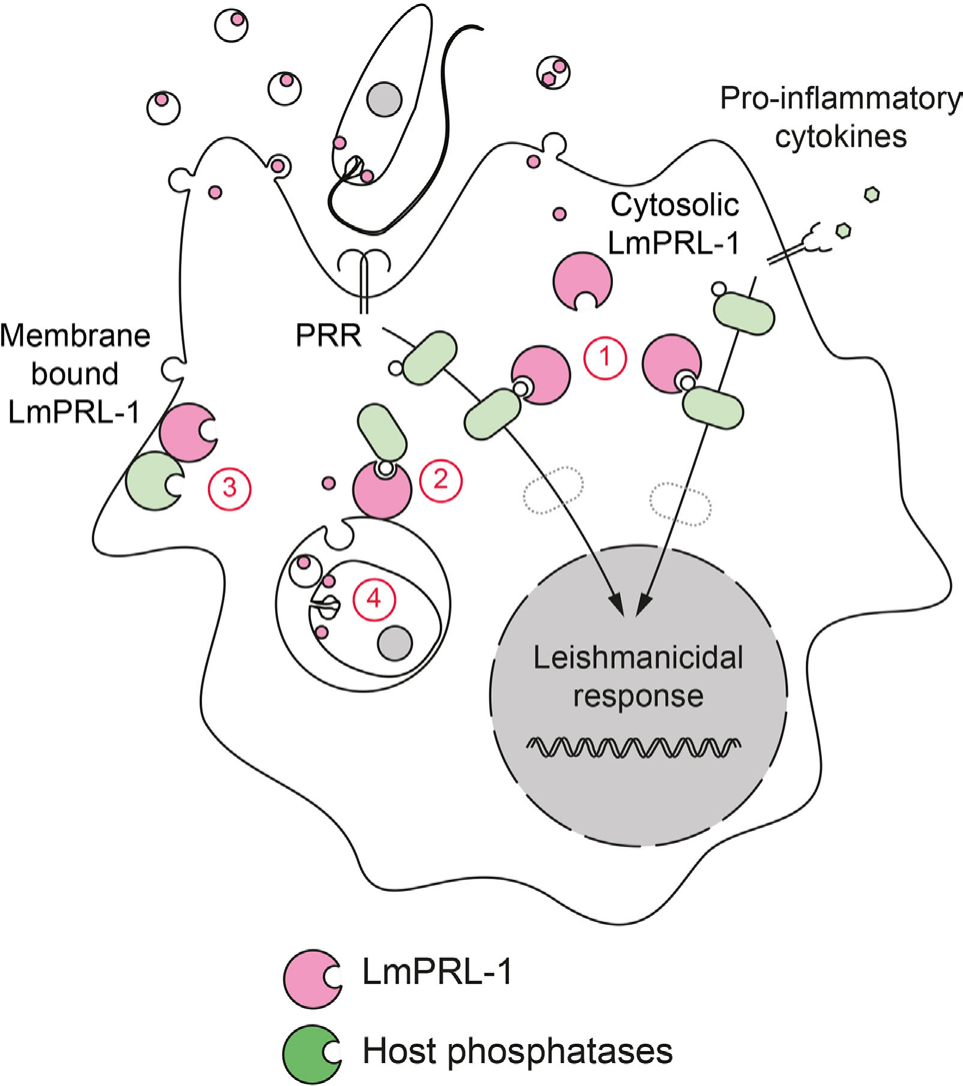
Hypotheses on the virulence mechanism of LmPRL-1. The phosphatase LmPRL-1 is secreted by *Leishmania major* promastigotes and amastigotes during macrophages infection. Once secreted, LmPRL-1 is localized at the membrane of the parasitophorous vacuole (PV) and/or in the cytosol of the infected macrophage. The observed support of intracellular parasite survival by LmPRL-1 could result from an inhibitory effect on important signaling pathways that otherwise promote the microbicidal response of the host macrophage (1). Other pathways not directly related to the innate immune response, but contributing to the general fitness of the macrophage and its function as host cell niche for the parasite could also be targeted by LmPRL-1 (2). Putative targets of LmPRL-1 are the host PRL proteins. Mammalian PRL activity relies on the formation of PRL trimers. LmPRL-1 might modulate the activity and function of its mammalian orthologs by physically interacting with them (3). Finally, a parasite-autonomous, intrinsic prosurvival effect of LmPRL-1 also needs to be envisaged (4).

Structurally, we verified that *Leishmania* PRL-1, like its mammalian homolog, has two important characteristics. First, its cysteine-based catalytic site was shown to be under the control of a neighboring regulatory cysteine. Under oxidative conditions, this cysteine formed a transient inactivating disulfide bridge with the catalytic cysteine. Second, a third cysteine at the C-terminus could be farnesylated. This posttranslational modification controlled the correct subcellular localization inside promastigotes. During metacyclogenesis, LmPRL-1 was constantly expressed by promastigotes. Its level tended to decrease after amastigotes transformation in macrophages and also showed a weaker expression in amastigotes isolated from footpad lesions of *L. major*-infected BALB/c mice. Strikingly, during the first days of macrophage infection LmPRL-1 reached the cytoplasm of its host cells through the exosome route. There, LmPRL-1 appeared to support the intracellular replication of the parasite in primary mouse macrophages (118). We currently do not know the molecular mechanism by which LmPRL-1 promotes *L. major* virulence. One possibility is that LmPRL-1 has an intrinsic regulatory effect on parasite development and/or transformation. As secreted LmPRL-1 is detectable in host cell cytosol, it is also plausible to assume that LmPRL-1 modulates host cell signaling pathways by targeting phosphoproteins, interfering with the normal function of host PRLs, or altering the activity of other secreted virulence factors (Figure 5). We currently address these possible mechanisms in studies with LmPRL-1-deficient *L. major* parasites.

## Concluding Remarks

Host–pathogen interactions taking place during leishmaniasis rely on complex antagonistic and synergistic molecular interplays some of which we have summarized in this review.

*Leishmania* parasite survival and disease development depend on the fitness of the host immune reaction. To counteract the generation of a microbicidal host response, *Leishmania* parasites have developed efficient strategies to extinguish or impede the proinflammatory signaling pathways triggered by their detection. Proteases secreted by the parasite, such GP63 and LmCPb, represent important components to achieve this goal and finally escape the deadly production of leishmanicidal compounds. Among the targets of these virulence factors, host protein phosphatases, especially SHP-1, are of particular relevance. Once proteolytically activated, these host phosphatases can directly inhibit the signaling pathways that are required for an effective anti-*Leishmania* host response (Figure 3).

While the parasite-driven activation of host phosphatases appears to be the *modus operandi* of choice for *Leishmania* to impede host immune responses, an additional strategy has been postulated. Already in the 1980s, the discovery of SAcP activities suggested that *Leishmania* parasites could use its own secreted enzymes to manipulate its host. However, until now a link between SAcP and *Leishmania* virulence has not yet been ascertained, probably because of the redundancy between the different orthologous gene encoded by each *Leishmania* genome. Only recently, our study of the *Leishmania* dual specificity phosphatase of the PRL family, LmPRL-1, provided the first evidence for a phosphatase secreted by *Leishmania* that promotes the virulence of the parasite during macrophage infection (Figure 5). As LmPRL-1 is secreted within exosomes, it is accompanied by a miscellaneous pool of proteins and small RNAs (174). Among these exosomal proteins, five other phosphatases have been detected that require further characterization with respect to their possible role as virulence factors of the parasite and their participation in the modulation of phosphorylation events in host cells.

In the past, the mechanism of activation of host phosphatases by *Leishmania* has been extensively studied by several research groups and thereby led to the identification of some of the signaling pathways that are targeted by the parasite. The efforts should now focus on the biochemical and functional characterization of new *Leishmania* virulence factors that directly interfere with phosphorylation events in host cells. In addition, our knowledge of host and *Leishmania* phosphatases that was mainly acquired in mouse models, still requires to be tested in clinical settings in order to establish whether differential expression of *Leishmania* phosphatases contributes to the severe courses of infection seen in mucocutaneous or visceral leishmaniasis.

## Author Contributions

All authors listed, have made a substantial, direct and intellectual contribution to the work, and approved it for publication.

## Conflict of Interest Statement

The authors declare that the research was conducted in the absence of any commercial or financial relationships that could be construed as a potential conflict of interest.

## Footnotes

^1^http://www.who.int/mediacentre/factsheets/fs375/en/.

^2^http://www.who.int/neglected_diseases/diseases/en/.
